# Unilateral Opening of Rat Blood-Brain Barrier Assisted by Diagnostic Ultrasound Targeted Microbubbles Destruction

**DOI:** 10.1155/2016/4759750

**Published:** 2016-08-04

**Authors:** Yali Xu, Hai Cui, Qiong Zhu, Xing Hua, Hongmei Xia, Kaibin Tan, Yunhua Gao, Jing Zhao, Zheng Liu

**Affiliations:** ^1^Department of Ultrasound, Xinqiao Hospital, The Third Military Medical University, 183 Xinqiao Street, Chongqing 400037, China; ^2^Department of Ultrasound, Southwest Hospital, The Third Military Medical University, Chongqing 400038, China; ^3^Department of Ultrasound, Sichuan Provincial Hospital for Women and Children, Chengdu, Sichuan 610000, China

## Abstract

*Objective*. Blood-brain barrier (BBB) is a key obstacle that prevents the medication from blood to the brain. Microbubble-enhanced cavitation by focused ultrasound can open the BBB and proves to be valuable in the brain drug delivery. The study aimed to explore the feasibility, efficacy, and safety of unilateral opening of BBB using diagnostic ultrasound targeted microbubbles destruction in rats.* Methods*. A transtemporal bone irradiation of diagnostic ultrasound and intravenous injection of lipid-coated microbubbles were performed at unilateral hemisphere. Pathological changes were monitored. Evans Blue extravasation grades, extraction from brain tissue, and fluorescence optical density were quantified. Lanthanum nitrate was traced by transmission electron microscopy.* Results*. After diagnostic ultrasound mediated microbubbles destruction, Evans Blue extravasation and fluorescence integrated optical density were significantly higher in the irradiated hemisphere than the contralateral side (all *p* < 0.01). Erythrocytes extravasations were demonstrated in the ultrasound-exposed hemisphere (4 ± 1, grade 2) while being invisible in the control side. Lanthanum nitrate tracers leaked through interendothelial cleft and spread to the nerve fiber existed in the irradiation side.* Conclusions*. Transtemporal bone irradiation under DUS mediated microbubble destruction provides us with a more accessible, safer, and higher selective BBB opening approach in rats, which is advantageous in brain targeted drugs delivery.

## 1. Introduction

Blood-brain barrier (BBB) remains a key obstacle in the delivery of medication into the brain parenchyma due to its physical barrier made of brain capillary endothelial cells, tight junction, extracellular base membrane, adjoining pericytes, astrocytes, and microglia [1]. Drug transport to the brain is hampered by this almost impermeable, highly selective, and well-coordinated barrier. More than 98% of all small-molecule drugs and approximately 100% of large-molecule neurotherapeutics are excluded from the brain by BBB [1]. Only small-molecule drugs with high lipid solubility and a low molecular mass under 400~500 Da can cross the BBB in pharmacologically significant amounts [2].

Some methods have been tried to disrupt BBB, such as modifying the agents to allow them to penetrate the BBB or using drug carriers such as liposomes and nanoparticles [3] or conjugating the drugs with a protein [4] or using an antibody to ferry drugs across the BBB [5]. However, only a finite amount of drugs can be delivered due to a limited number of receptors and a discrete quantity of molecules can be attached to a carrier. BBB has been isolated as the rate-limiting factor in brain drug delivery. So it is of great clinical interest and importance to explore an applicable method to open the BBB effectively, noninvasively, and selectively to facilitate the drug or stem cell delivery into brain [6, 7].

The advent of ultrasound mediated microbubbles destruction assisted BBB opening provided a new insight in drug delivery into the brain [8]. This approach was firstly developed by applying a focused ultrasound system (FUS) combined with a circulating ultrasound contrast agent (Optison) [8]. Similar studies were reported in the following years and the effectiveness [9–23], safety [9, 10, 13, 20–22], and targeting [7, 8, 16–18, 21] of BBB opening were proved via this method. Diagnostic ultrasound (DUS) is much safer and more accessible than therapeutic ultrasound, but only a few studies were designed to use DUS mediated microbubbles system on BBB disruption [24–27]. Our research team have investigated the impact of the intensity/power and irradiation duration of DUS, the dosage of microbubble on BBB permeability, and the possible mechanism on BBB opening [24–26]. Since the investigations on DUS assisted BBB disruption remain limited, the present study used DUS irradiated lipid-coated microbubbles destruction on intact rat temporal bone, aiming to explore the feasibility, efficacy, and safety of DUS mediated microbubble system on unilateral BBB opening and to facilitate the targeted delivery of therapeutic agents into the brain in further studies.

## 2. Materials and Methods

### 2.1. Ethics Statement

This study was carried out in strict accordance with the recommendations in the Guide for the Care and Use of Laboratory Animals of the National Institutes of Health. Animals in this study were provided by the Experimental Animal Center of the Third Military Medical University, with the quarantine license number of SCXK (Chongqing) 2007-0003. All the procedures were approved by Chongqing Ethics Committee, and all the experiments were performed in accordance with guidelines from the Chinese Animal Welfare Agency.

### 2.2. Animal Preparation

The study subjects were male SD rats (*n* = 30), weighed 180~220 g. Intraperitoneal injection anesthesia was performed at a dosage of 20 mg/Kg of 2% amyl sodium pentobarbital, and the rats were placed at a prone position.

### 2.3. Diagnostic Ultrasound System

A GE Vivid 7 diagnostic ultrasound apparatus was used with an adult echocardiogram detector (M4S), a wide-band, multifrequency active matrix phased array probe with a beam-width of 28*∗*20 mm and 90 degree field of view, which supports cardiac and transcranial applications, and the parameters acquired from the manual were set as follows: harmonic frequency, 1.9/3.3 MHz; mechanical index (MI), 1.3; peak negative pressure amplitude (PNP), 1.82 MPa; pulse duration, 0.84 ms; PRF, 2567 Hz; real-time contrast imaging mode; focus depth, 3 cm; continuous exposure duration, 10 min.

### 2.4. Lipid Microbubbles

A lipid-coated microbubbles contrast agent, named Zhifuxian, was awarded a National Invention Patent by China in 2005 and used for the nucleation of ultrasound cavitation. Zhifuxian was prepared by lyophilisation of suspension of two lipids, 1,2-dipalmitoyl-sn-glycero-3-phosphoglycerol and 1,2-Distearoyl-sn–glycero-3-phosphoethanolamine, and agitated with perfluoropropane gas (Changluhuaxin Co. Ltd., Tianjin, China) using a high-speed mechanical amalgamator [28]. The characteristics of Zhifuxian are as follows: mean particle diameter 2 *μ*m, size distribution (1~5) *μ*m, and bubble concentration (4~9) × 10^10^/mL. Zhifuxian enhanced ultrasonic effect has been used in animal studies in our Lab [29–31].

### 2.5. Experimental Procedure

A total of 30 SPF (specific pathogen free) rats were used in the experiment, 25 for Evans Blue (EB) extravasation and pathological examination and 5 for BBB observation under transmission electronic microscopy (TEM). All the invasive procedures were preceded by an adequate anesthesia.

**Figure 1:**
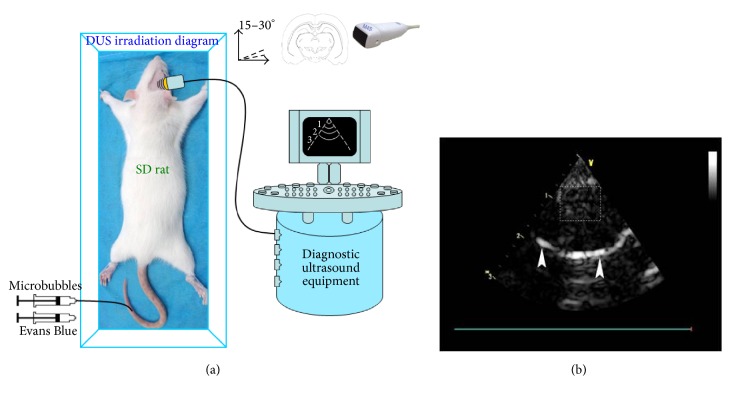
Diagrams of transtemporal exposure with DUS and brain perfusion image with microbubbles in rats. (a) The long axis of the M4S probe was kept consistent with the connection line of the right eye and ear of rat, and a tilting position was fixed with an angle of 15° ~ 30° to the horizontal plane during the exposure. (b) Brain parenchyma was mildly enhanced after microbubbles infusion in rats (white box indicates the perfusion of the targeted area and white arrowheads indicate the skull of the contralateral side of rat).

In the procedure of diagnostic ultrasound (DUS) exposure, the ultrasound probe was set at the right side of the temporal bone with the scanning plane consistent to the connection line between the right eye and right ear. The probe was kept at tilting position with an angle of 15° ~ 30° to the horizontal plane (Figure 1(a)). Before DUS irradiation, the rat brain was observed with two-dimensional ultrasound and brain perfusion imaging modality after intravenous infusion of 0.1 mL microbubbles to make sure the ultrasound beam is within the targeted brain. Then the probe was fixed manually during the 10 min exposure. All the contralateral brain hemispheres were not treated and used as the controls. The lipid microbubbles were injected via tail vein at a dose of 200 *μ*L once the DUS exposure was initiated, followed by a bolus injection of 50 mg/Kg 2% filter-sterilized solution of EB and 1 mL saline to wash the tube. Brain perfusion images were visualized and recorded to ensure that the rat brain was constantly within DUS irradiation field. The rats were sacrificed under anesthesia 1 h after exposure, the thoracotomy was performed, and a catheter was inserted via the left ventricular cannulation into the aorta. The heart was rinsed by heparinized saline with a pressure of 100~120 cm H_2_O (1 cm H_2_O = 98 Pa) until the liquid coming out of the right atrium became clear, and the craniotomy was performed to take out the brain.

### 2.6. Macroscopic Observation of the Brain Specimen

EB extravasation in the brain was observed at the general view of the gross brain specimen. And then, the brain was cut into 3 equal parts (parts A, B, and C) at coronal plane segmentation: part A for quantification analysis of EB extraction and semiquantification of EB extravasation, part B for quantification of EB FIOD under LCM, and part C for histological examination and erythrocyte extravasation analysis.

### 2.7. Semiquantitative Grading of EB Extravasation

The coronal plane segmentation of part A brain parenchyma was cut into 5 slices with a thickness of 4~6 mm and analyzed with EB extravasation grading, according to the standard of Table 1, and was compared between the DUS-exposed and the control hemisphere.

### 2.8. Quantitative Analysis of EB Extraction from Brain

Brain tissues at the hippocampus and cortex of both the DUS-exposed and the contralateral hemispheres (with the same weight around 2.5 g) were weighed, cut into pieces, and immersed in formamide liquid at 37°C water bath for 24 h. EB solution was extracted from the brain tissue and its optical density was analyzed by a spectrophotometer (DU800 UV) at 620 nm, in which 5 *μ*g/mL was set as the initial concentration and multiple proportional dilution was used, with a gradient solution of 5, 2.5, 1.25, 0.625, 0.3175, and 0.15875 (*μ*g/mL) for the EB standard concentration to obtain the regression equation *y* = 23.904*x* − 0.011 (*y*, EB concentration, and *x*, the optical density), with the coefficient of determination *R* ^2^ = 0.9995, *F* = 7498.723 (*p* < 0.001). The content of EB in per gram of brain mass was obtained and compared between the DUS-exposed hemisphere and the control.

### 2.9. Quantification of the EB under LCM

Three frozen slices from brain hemispheres of part B were selected, with a slice thickness of 30 *μ*m and every 3 mm between each slice. Red light was set and the photos were taken with laser confocal microscopy (LCM). The red fluorescence integrated optical density (FIOD) values of EB in the DUS-exposed and contralateral hemispheres of the same area (3.3 mm^2^) were measured using Image-Pro Plus software and statistically analyzed. The scale of the FIOD reflects the deposition of EB in brain tissue.

### 2.10. Histological Examination

Coronal slices of brain tissue of part C were fixed with 10% formalin, paraffin-embedded, and stained with H&E, and histological changes including blood cell extravasations in brain tissue were observed under light microscope. Five sample areas of the same location of the DUS-exposed and the control hemispheres were observed, with each sample of 5 slices, and the pathological changes of vessel injuries and erythrocyte extravasation were graded according to the standard of Table 2. An extravasation was defined as ≥2 red blood cells in the parenchyma adjacent to an intact or partially intact blood vessel. All the continuous slices of the brain samples were observed and the extravasation sites were counted in the two hemispheres.

### 2.11. Assessment of BBB Permeability under TEM

 Five rats that undertaken the same procedure were used for Lanthanum nitrate tracing under TEM. After aortic infusion for 5 min with heparinized saline, 1 mm^3^ of the brain samples at the same part of two hemispheres was fixed at 4°C overnight with 0.1% glutaraldehyde, 2% Lanthanum nitrate, 2% polyformaldehyde, and 3% sodium cacodylate perfusion. Specimen of the brain tissue was washed twice with Lanthanum-containing cacodylate buffer, fixed with Lanthanum-containing 1% osmium tetroxide. After acetone gradient dehydration and Epon 812 embedding, thin slices were cut with microtome (LKB4800A, Microm, Germany) and observed under electron microscopy (TECNAI-10, FEI, Netherland).

### 2.12. Statistical Analyses

The extraction, FIOD, and extravasation grading of EB and erythrocytes extravasation grading and sites number at two hemispheres of rats were described as means ± SD, and paired samples* t*-test (continuous data) and nonparametric two related sample tests (categorical data) were used to analyze the variables between the DUS-exposed and the contralateral hemisphere. All the analyses were performed with SPSS 13.0 (SPSS Inc., Chicago, USA). All *p* values less than 0.05 were considered statistically significant.

## 3. Results 

### 3.1. Animal Survival

Five rats died during the anesthesia and the experiment procedure and 25 rats survived and were included for analysis, with 20 for histological examination and EB quantification and 5 for TEM examination. 

### 3.2. Brain Perfusion Imaging after Intravenous Infusion of Microbubbles

The rat brains were observed under two-dimensional ultrasound and brain perfusion images were recorded to insure the targeted irradiation of brain under ultrasound beam. The rat brain parenchyma is in hypoecho with the skull in a hyperechogenic arc, which indicated that both the irradiated and the contralateral sides were under the ultrasound beam, and the irradiated hemisphere is located at the near field of ultrasound beam. After microbubbles infusion, the brain parenchyma is mildly enhanced (Figure 1(b), white arrows indicate the skull of contralateral side).

### 3.3. Semiquantification Analysis of EB Extravasation Grading

**Figure 2:**
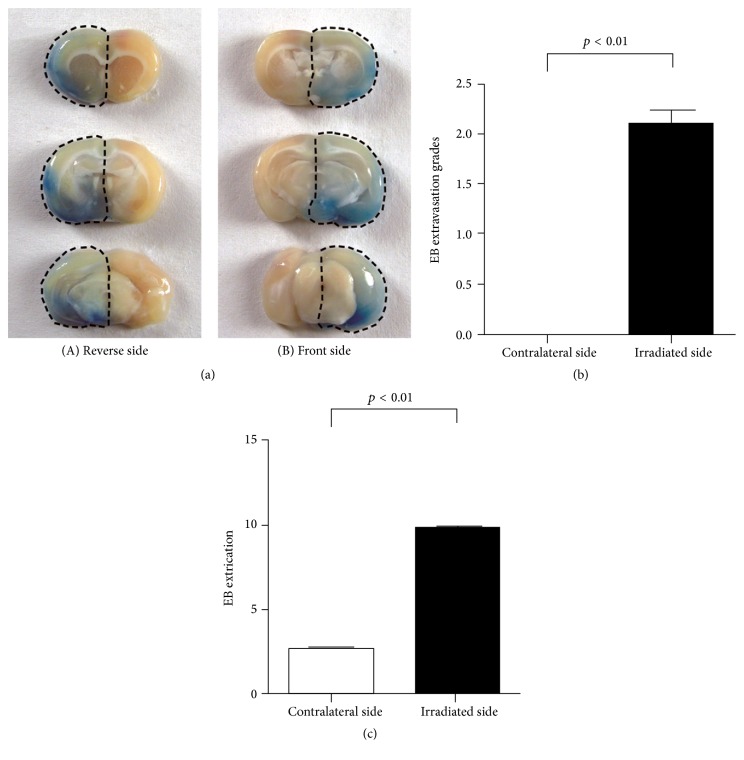
EB extravasation of brain parenchyma. (a) A substantial EB extravasation was observed at the coronal slices (front and reverse sides) of the DUS-exposed hemisphere while it is not visible at the contralateral side. (b) Histogram of the quantification analysis of the EB extravasation grading: a significantly higher grade was indicated in the DUS-exposed hemisphere than the contralateral hemisphere (*p* < 0.01). (c) Histogram of the quantification analysis of the EB extraction from the brain: a significantly higher value was shown in the DUS-exposed side compared to the contralateral side (*p* < 0.01).

A patchy distribution of EB extravasation was observed at the DUS-exposed hemisphere at continuous slices of coronal view and located at the hippocampus, cortex, corpus callosum, and thalamus (Figure 2(a)), while being not visible at the contralateral in both gross appearance and coronal slices of brain parenchyma. The EB grades at the irradiation hemisphere were significantly higher than that at the contralateral side (2.29 ± 0.10 versus 0, *Z* = −3.944, *p* < 0.01, and *n* = 20 and 5 slices of each specimen, Figure 2(b)).

### 3.4. Quantitative Analysis of EB Extraction from Brain Tissue

The EB extracted from brain tissue of the DUS-exposed hemisphere was much more than that from the contralateral side (9.85 ± 0.75 versus 2.69 ± 0.72 *μ*g/g, *p* < 0.01, Figure 2(c)).

### 3.5. Quantitative Analysis of EB FIOD in Brain Parenchyma

**Figure 3:**
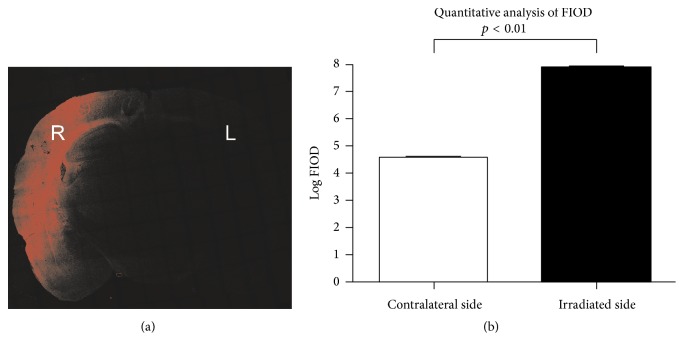
Red FIOD of coronal brain tissue under LCM. (a) Substantial red fluorescence was found at the DUS-exposed hemisphere (R) compared to the control (L). (b) Log FIOD in the DUS-exposed side was significantly higher than that of the contralateral side (*p* < 0.01).

FIOD was measured at the frozen sections of coronal brain tissue under LCM, Figure 3(a). FIOD was significantly higher at the DUS-exposed hemisphere than that of the control (7.72 ± 0.76 × 10^7^ versus 3.45 ± 1.10 × 10^4^, *p* < 0.01, *n* = 20 and 3 slices of each specimen); the difference in Log FIOD between the ultrasound treated and the control was also significant (7.89 ± 4.88 versus 4.54 ± 4.00, *p* < 0.01, Figure 3(b)).

### 3.6. Erythrocytes Extravasation Grading and Distribution in Brain Tissue after DUS Irradiation

**Figure 4:**
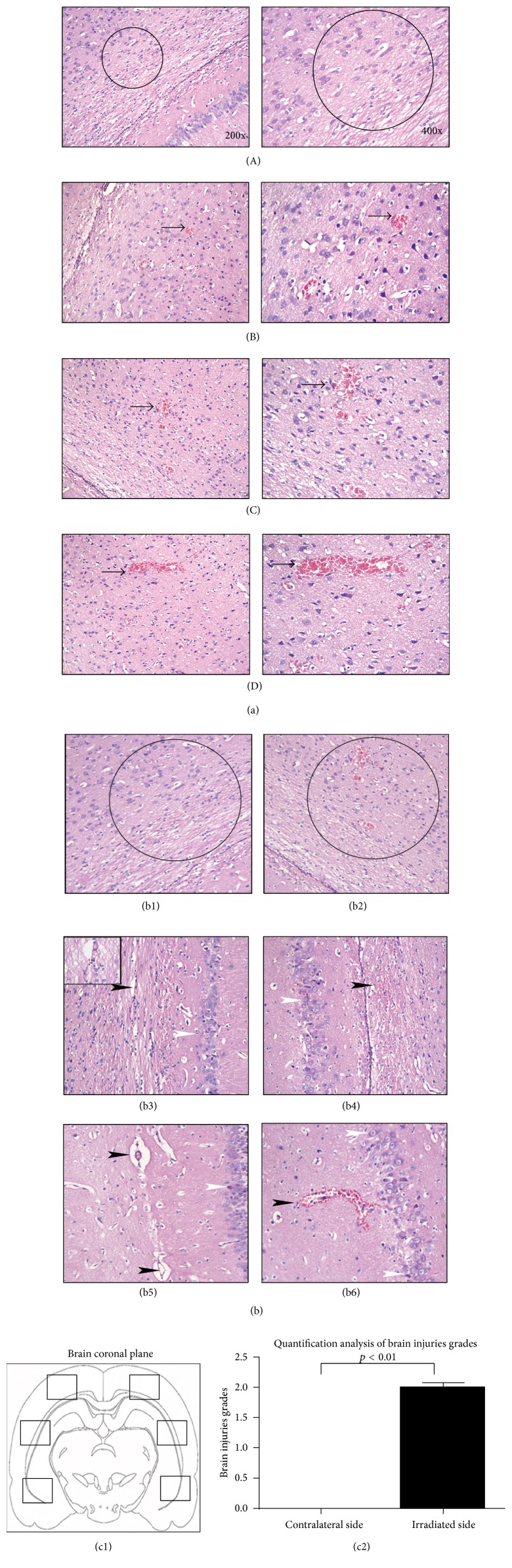
Histological changes after DUS exposure in brain parenchyma. The cerebral vascular space was widened with visible leakage of red blood cells by punctate or small pieces distributed evenly in the cortex, corpus callosum, and hippocampus at the DUS-exposed hemisphere under light microscope ((a) left 200x and right 400x). Erythrocytes extravasation grading: (A), 0; (B), 1; (C), 2; (D), 3; black arrows indicated erythrocyte extravasation; significantly higher erythrocytes extravasation grade was noted in the histogram at the DUS-exposed hemisphere compared to the control (c2), (c1) diagram of the observation locations in brain parenchyma, (b): (b1)–(b6) pathological changes at the DUS-exposed and contralateral hemispheres (400x): cortex ((b1) contralateral side and (b2) DUS-exposed side), corpus callosum ((b3) contralateral side and (b4) DUS-exposed side); and hippocampus ((b5) contralateral side and (b6) DUS-exposed side); black arrowheads, erythrocytes extravasations.

Histological changes were observed and extravasation grading was analyzed with H&E staining slices. The erythrocyte extravasation grading was shown in Figure 4(a), left 200x and right 400x. The erythrocytes leakage was distributed evenly with punctate or small pieces in the cortex, corpus callosum, and hippocampus (Figures 4(b)). The extravasation grades at the DUS-exposed hemisphere were significantly higher than those at the contralateral side (2.01 ± 0.30 versus 0, *Z* = −4.089, *p* < 0.01, and *n* = 20 and 5 slices of each specimen, Figure 4(c)). The mean value of erythrocyte extravasation sites was 4 ± 1 in the DUS-exposed hemisphere versus 0 in the contralateral side in part C brain specimen. The histological changes at the irradiated brain hemisphere: the cerebral vascular space was widened with normal morphology, intact nucleolus, and integrated nuclear membrane in nerve cells; no visible injuries were found in neurons; neither any erythrocyte leakages nor tissue damages were observed at the contralateral hemisphere (Figure 4(b)).

### 3.7. Enhancement of the BBB Permeability after DUS Irradiation under TEM

**Figure 5:**
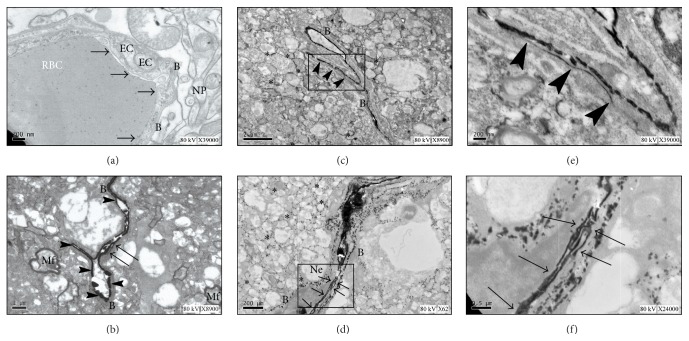
Nitrate Lanthanum distributions after ultrasonic irradiation under TEM. (a) Normal structure of endothelial cell (EC) layer and basement membrane (B) of BBB (black arrow) after Lanthanum injection in the control rat; NP: neuropil. (b) Findings in the contralateral hemisphere. Lanthanum ions were located within the capillary wall and distributed along the endothelial cell layer (arrowheads); a small amount of Lanthanum leaked through the gap of endothelial cell and stopped at the first tight junction (black arrows); Mf: medullated fibers. (c-d) Findings in the irradiated hemisphere. (c) Nitrate Lanthanum leaked through the interendothelial cleft at a whole length (arrowheads), crossed the basement membrane (B), and infiltrated to the glial tissue gap (asterisks); Mf: medullated fibers. (d) Lanthanum leaked extensively through the interendothelial cleft at a different vessel (arrows) and diffused to the gap of nerve fibers (asterisks), and mild swelling of the neurons close to the vessel was indicated. Ne: neurons (scale bars: (a) = (d) = 200 nm, (b) = 1 *μ*m, and (c) = 2 *μ*m). (e) High magnification image for the box region in figure (c). (f) High magnification image for the box region in figure (d).

The distribution of Nitrate Lanthanum in the hemisphere of the DUS-exposed rat (*n* = 4) and the blank control rat (*n* = 1, without ultrasonic exposure) was demonstrated under TEM (Figure 4). Normal structure of endothelial cell (EC) layer and basement membrane (B) of BBB after Lanthanum injection was shown in the control rat (Figure 5(a)). Lanthanum ions were located within the vessel wall, distributed along the endothelial cell layer and a small amount leaked through endothelial cell, and stopped at the first tight junction (Figure 5(b)); Nitrate Lanthanum leaked through the interendothelial cleft at the whole length of vessel, crossing the basement membrane (B) and infiltrating to the glial tissue gap (Figure 5(c)); an extensive leakage of Lanthanum crossed through the interendothelial cleft at a different vessel and diffused to the gap of nerve fibers, and mild swelling of the neurons close to the vessel was indicated (Figure 5(d)).

## 4. Discussion

In this study, an applicable and commercialized DUS was performed on rat temporal skull combined with intravenous injection of self-made lipid microbubbles to open the BBB unilaterally and the results showed that it was a useful method to increase the BBB permeability successfully, selectively, and noninvasively.

### 4.1. Effective and Unilateral Opening of the BBB

A transskull irradiation on the temporal lobe of rats under DUS mediated microbubble system has successfully facilitated the unilateral BBB opening mainly located at the hippocampus, cortex, corpus callosum, and thalamus, and the histological changes were found at the same area as well, which not only exert locally and selectively opening of the BBB within the irradiated hemisphere but also prove to be promising in the targeted delivery of therapeutic agents into CNS diseases unilaterally located such as brain glioma and brain ischemia. Compared to the contralateral hemisphere, significant differences were observed in the DUS-exposed hemisphere in EB (with a molecular weight of 960.80 Da) leakage, EB extraction, and Lanthanum ion (with a molecular weight of 433 Da) leakage as well.

The previous studies performed by our lab have demonstrated a successful opening of the BBB under DUS mediated microbubbles destruction under the same procedure but at different exposure angle. Ultrasound probe (M4S) was put to the middle position between the right eye and ear, and the detector was parallel to the horizontal plane. The permeability of BBB was increased in both hemispheres with substantial changes in the irradiated hemisphere [2]. The present study was aimed at opening the BBB unilaterally for further studies. We tried different acoustic windows and irradiation angles; for different window, a transskull irradiation on the vertex was tried and EB extravasations were distributed at both sides of brain; for different angle, the ultrasound detector was placed at the temporal bone and a parallel irradiation at the horizontal plane was also tried, and minimal EB extravasation at the contralateral side was demonstrated. Only by the way we have described, we can get the result of unilateral opening of BBB mainly located at the hippocampus, cortex, and corpus callosum of the irradiated hemisphere. Some key factors were explored on the targeted opening of unilateral BBB to put the probe to the temporal lobe (exactly located in the middle position of the connection line between the ear and eye); to keep the probe at a tilting angle of 15° ~ 30° to the horizontal plane; and to set the focus depth of DUS at 3 cm. It is know that DUS is more difficult than FUS on the targeted opening of BBB due to the unfocused ultrasound beam, but the present study has found out that an unilateral and local opening of the BBB was successfully demonstrated under commercially available DUS irradiated microbubbles system even without the guidance of other imaging equipment such as MRI, which brought a promising future for the targeted delivery of medications to hippocampus-centered diseases such as Alzheimer's disease. The unilateral opening of BBB might be caused by the attenuation of rat skull especially at the far field of the contralateral brain hemisphere, and a lower or minimal ultrasonic effect and lesser effect on BBB disruption was produced at the contralateral side compared to the insonated hemisphere.

### 4.2. Noninvasive and Safe Opening of the BBB under DUS Mediated Microbubbles Destruction

Erythrocytes extravasation was defined as ≥2 red blood cells in the parenchyma adjacent to an intact or partially intact blood vessel, and seven or less extravasations were considered minimal brain injury [27]. The present study proved to be a noninvasive method with mild erythrocytes extravasations (an average of 4 sites in 1/3 of the exposed right brain hemisphere and 12 extravasations per brain sample and 2 in erythrocytes extravasation grading) and minimal injury on neurons under electronic microscopy. Bing et al. [27] reported in a preliminary study that a standard B-mode ultrasound exposure (MI = 1.3) combined with Definity was found to open wide planes of BBB corresponding to the B-mode field of view and to be associated with blood cell extravasation (26 sites in one brain). Hynynen et al. [8] have reported that a focused ultrasound system with a frequency of 1.63 MHz induced a reproducible and reversible disruption of the BBB and a pressure amplitude level ≥ 2.3 MPa would cause necrosis in 70~80% of the irradiated brain area and neurons loss, while a value below 2.3 MPa only brought a small amount of erythrocytes extravasations. Compared to these studies less injuries were produced in brain in the present study and the possible reasons were described in the following: a DUS irradiation system with lower intensity was tried instead of therapeutic one; a lower DUS PNP (maximum value of 1.82 MPa within the acoustic field) was chosen in the transtemporal irradiation in rats; self-made lipid microbubble (Zhifuxian) might have poorer stability and stimulates lower effect in BBB disruption than Definity.

This transintact skull irradiation system is not only a successful but also a noninvasive method to open the BBB without substantial impairment to the skull and brain hemisphere in small animals. Some middle-size animals (New Zealand rabbits) have been tried in our group as well; since the detector is available for adult cardiac image, it is promising and feasible for big animals and human trials; more studies should be tried in big animals and human studies in the future. But the safety of transcranial ultrasound especially in the presence of microbubbles needs to be paid attention on clinical examinations.

### 4.3. Limitations of the Study

Due to its big scale and volume, the DUS is too large to be transported for the detection of the real acoustic and cavitation parameters; a portable DUS might be a better choice to have these important parameters detected in the future studies. A long exposure duration (10 min) and high MI (1.3) was used in the study, and a shorter exposure time and lower MI should be tried to explore the efficacy and safety in the future; the reversibility of the enhancement of BBB permeability was not studied; mild hemorrhage was elicited in the exposed brain regions; a mechanical positioning system with a fixed device should be set up for experimental arrangement.

## 5. Conclusions 

Transtemporal bone irradiation under DUS mediated microbubble destruction provides us a more accessible, safer, and higher selective BBB opening approach in rats, which is advantageous in targeted delivery of drugs into cerebrovascular diseases.

## Figures and Tables

**Table 1:** EB extravasation grading in brain hemisphere under DUS irradiation.

Grading	Description
0	No apparent EB extravasation
1	A small amount of EB extravasation within the cerebral cortex
2	Flake dispersion of EB at the lateral ventricle or hippocampal layer
3	Massive EB extravasation in the brain tissue

**Table 2:** Erythrocyte extravasation grading in brain parenchyma under DUS irradiation.

Grading	Description
0	Almost no capillary injury and erythrocyte leakage
1	Several or a trace amount of erythrocyte extravasations
2	Petechial or sheet erythrocyte extravasations
3	Heavy bleeding or infarction lesions
